# Parachute-like pull-through anastomosis for low rectal cancer: a new method for preservation of anal function

**DOI:** 10.1007/s00423-023-02768-w

**Published:** 2023-02-13

**Authors:** JianWei Wang, Xun Ye, Qin Zhou, ChengCai Xu, YiQun Fan, Na Luan, XiaoLing Zhu

**Affiliations:** 1 Department of Colorectal Surgery and Oncology, Key Laboratory of Cancer Prevention and Intervention, Ministry of Education, 2nd Affiliated Hospital, Zhejiang University School of Medicine, Jiefang Road 88th, Hangzhou, China; 2 Department of Surgery, 4th Affiliated Hospital, School of Medicine, International Institutes of Medicine, Zhejiang University, Yiwu, China

**Keywords:** Rectal cancer, Pull-through, Coloanal, NOSES, Laparoscopy

## Abstract

**Background:**

With recent improvements in surgical technique, oncological outcomes of low rectal cancer have improved over time. But the QoL impairment as a result of anal functional disorder cannot be ignored. And the incidence of anastomosis-related complications cannot be ignored. To address these problems, a personal technique for pull-through coloanal anastomosis (parachute-like intussuscept pull-through anastomosis) was introduced and evaluated. This technique can relatively reduce surgical complications, minimize the impact of anal function, and obviate a colostomy creation.

**Methods:**

Between June 2020 and April 2021, 14 consecutive patients with rectal cancer underwent laparoscopic-assisted resection of rectal cancer in our hospital. Parachute-like pull-through anastomosis method was performed in all patients. Anal function, perioperative details, and postoperative outcomes were analyzed.

**Results:**

The mean (SD) operative time of first stage was 282.1 min (range 220–370) with an average estimated blood loss of 90.3 mL (range 33–200). And the mean (SD) operative time of second was 46 min (range 25–76) with an average estimated blood loss of 16.1 mL (range 5–50). Wexner scores declined significantly during the median follow-up of 18 months. Four postoperative anastomosis-related complications occurred in 14 patients, including perianastomotic abscess: 1 case (7%), anastomotic stricture: 1 case (7%), and colonic ischemia of the exteriorized colonic segment: 2 cases (14%).

**Conclusion:**

The results suggest that the method can facilitate safe and easy completion of coloanal anastomosis, using parachute-like pull-through anastomosis, with acceptable anal function.

## Introduction

Recently, the incidence of lower rectal carcinoma has significantly increased worldwide. Low rectal cancer is usually defined as the lower third of the rectum, within 6 cm from the anal verge [1]. Low rectal cancers were treated by a conventional coloanal anastomosis (CAA) [2] and intersphincteric resection (ISR). However, CAA is associated with a high rate of anastomotic leakage and pelvic sepsis [3, 4] which leads to the creation of ileostomy. In particular, patients with locally advanced very low rectal cancer typically receive neoadjuvant radio-chemotherapy (RCT) followed by a low coloanal or rectal anastomosis to guarantee a circumferential margin and R0 distal. Indeed, the fistula rate of low rectal anastomosis is still reported to be about 11%, even when a diverting ileostomy is created [5]. Nevertheless, studies have shown that the complication rates of temporary ileostomy were as high as 43%, including readmissions, dehydration, and chronic renal failure [6, 7]. Additionally, the construction of a stoma can impact the quality of life.

In 1939, Babcock [8] modified the Hochenegg [9] procedure and proposed a two-stage trans-anal colonic pull-through technique, but it did not receive much attention. Bacon [10], in 1945, re-proposed and popularized the operation, which is called Bacon operation. Due to the excision of the levator ani and the internal sphincter, the postoperative anal function is poor, and complications are numerous. In 1952, Black [11] modified the operation on this basis with retaining the levator ani and the internal sphincter, which significantly improved the anal function and reduced occurrence infection. Only during the healing process did anastomotic union occur. In 1961, the Turnball-Cutait delayed coloanal anastomosis was introduced, which also is a pull-through procedure [12, 13]. Studies suggest that the rate of anastomotic leakage of this method is visibly reduced [14], which may due to the adhesions between the anal canal wall, colonic serosa, and pelvic tissues that grow between the first and second stage [13]. Though the postoperative complications of the method are relatively rare, the results are comparatively severe. These complications include perianastomotic abscess, anastomotic stricture, colonic ischemia, and necrosis of the exteriorized colonic segment [14, 15]. In addition, the method overcomes the necessity of diverting ileostomy creation. But the recovery of bowel function is not ideal [16].

In order to reduce the incidence of these complications and improve the patients’ defecation functions, we designed a new pull-through colorectal anastomosis technique, including the colon is pulled through the anus after cut-off of internal anal sphincter in the first surgical stage, then reconstruction of the valve of Houston and the pelvic floor fascia; the pulled-through colonic segment was excised and the internal anal sphincter was repaired in the second stage.

We name the procedure the parachute-like intussuscept pull-through anastomosis (PIPA) and present the results of this initial study.

## Patient selection

Since 2020, we considered for a laparoscopic LAR with TME and delayed a “parachute” intussuscept coloanal anastomosis in patients fulfilling the following criteria:

1. Patients with carcinoma in the lower third of the rectum whose distance is less than 2 cm from the anal dentate line, or within 5 cm from anal verge
2. No invasion of internal sphincter and/or the levator ani muscle at imaging.
3. Frail patients have a high risk of anastomotic complications, such as after neoadjuvant therapy
4. Patients who refuse creation of a diverting or permanent stoma

## Surgical technique

### First stage

#### Abdominal phase

**Fig. 1:**
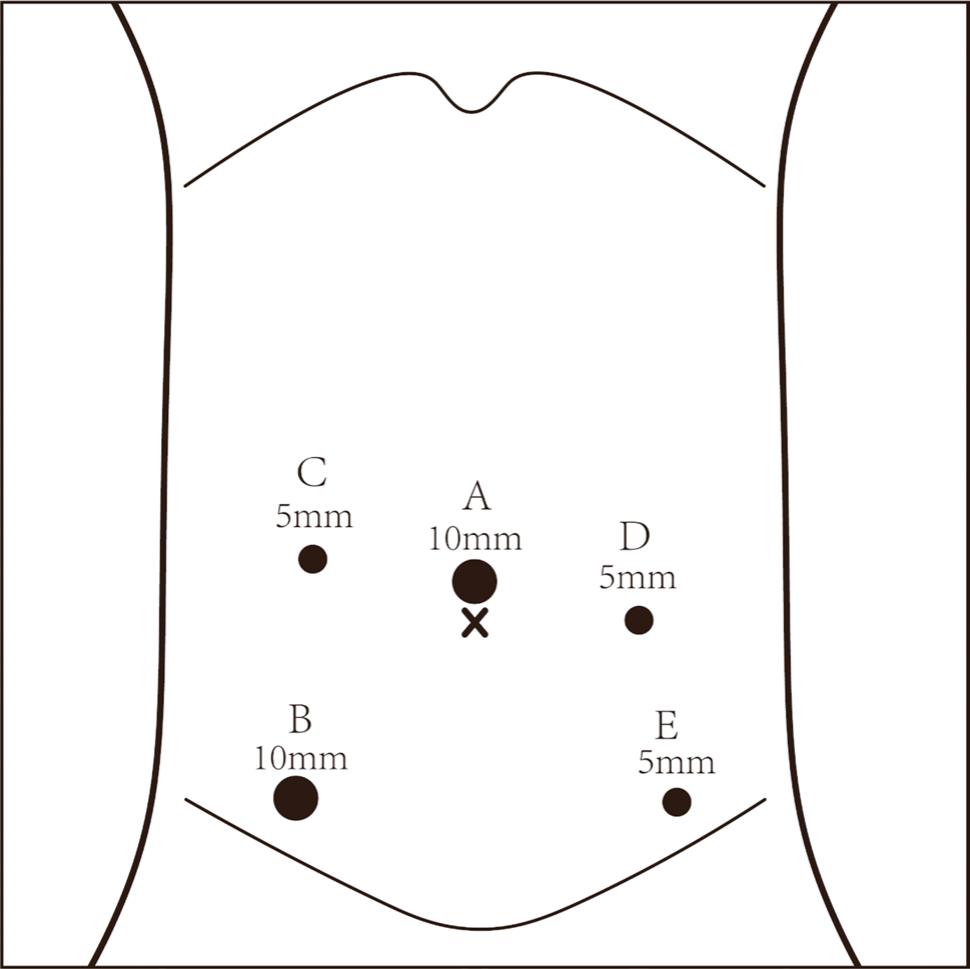
Trocar placement. **A** Camera port for laparoscopy. **B** Manipulation port for the surgeon. **C**, **D E** Assisted ports

After anesthesia, the patient was positioned in the lithotomy position; pneumoperitoneum was established at 10 ~ 12 mm Hg, and 4 trocars were placed in standard positions (Fig. 1). Surgery was performed according to oncologic principles of no-touch technique, performing low anterior resection (LAR) with total mesorectal excision (TME).

Briefly, the first step was adequate mobilization of the sigmoid colon up to the splenic flexure and complete dissection of the mesocolon. Following inferior mesenteric vessels were isolated and ligated, *mobilization* of the rectum and the mesorectum was performed. It was 2 cm under lower margin of tumor the anorectal junction was transected. In all cases, a rectal frozen section was collected and examined intraoperatively. In all cases, a margin frozen section was collected and examined intraoperatively. Particularly, part or all of the anococcygeal ligament should be retained as far as possible.

**Fig. 2:**
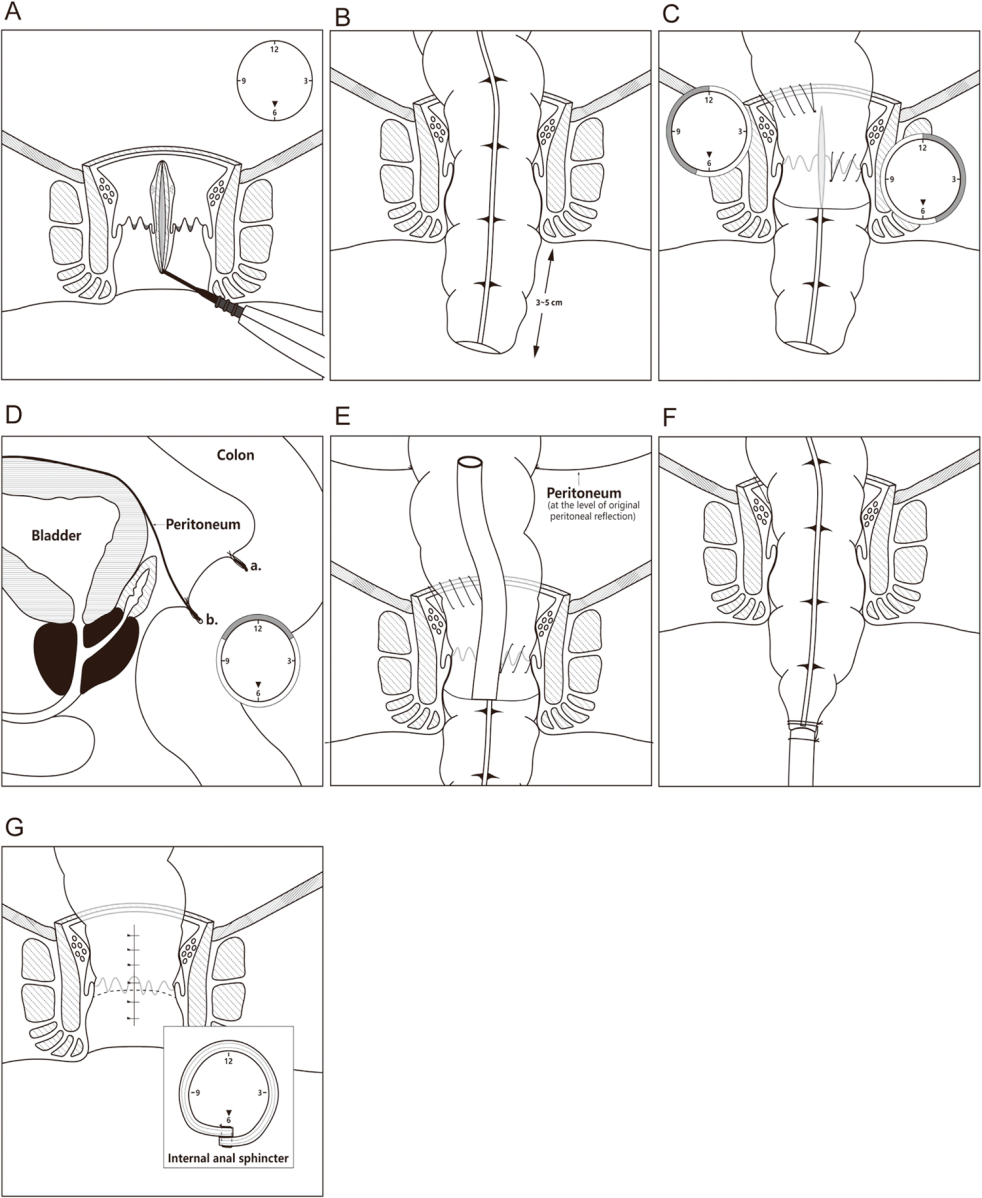
Surgical technique. **A** Total cut-off of internal anal sphincter was performed at the direction of 6 o’clock. **B** The rectum is pulled through the anus leaving approximately 3–5 cm of redundant colonic stump. **C** At the upper end of the rectal stump, the right row (from 7 to 12 o’clock) of continuous running sutures was placed between the rectal mucosal layer and all layers of the colon. Then, the left anterior row (from 12 to 5 o’clock) was also sutured by the same parachuting method as the right row using another stitch at the level of the dentate line. **D** Lambert’s suture was performed between anterior pelvic floor fascia and adjacent colon wall so that the colonic mucosa folds in the intestine form a valve, a reconstituted valve of Houston. Another valve of Houston was constructed 5 cm above the first one. **E** An endotracheal tube was inserted through the exteriorized colonic segment, with the end of the tube reaching the level of the reconstructed peritoneal reflection. **F** The tracheal catheter was sutured with exteriorized colonic segment. **G** The internal anal sphincter was repaired by folding and suturing both ends, and the anal was reconstructed

Lambert’s suture was performed between anterior pelvic floor fascia and adjacent colon wall so that the colonic mucosa folds in the intestine to form a valve, a reconstructed valve of Houston. Another valve of Houston was constructed 5 cm above the first one. Then, the pelvic floor fascia was closed, and the rectal retroflexion was reconstructed (Fig. 2D).

#### Perineal phase

After that, the anal orifice was gently enlarged until 3–4 fingers can enter, then a retractor was placed, and the dentate line was identified. At the direction of 6 o’clock, the mucosal area of rectal stump was incised instead of stripped, and total cut-off of internal anal sphincter was performed (Fig. 2A).

After laparoscopic verification of no tension, the proximal colon (sigmoid or descendens) was pulled through the anus leaving approximately 3–5 cm of redundant rectal stump. At the same time, the mesentery was aligned at the incision of the internal anal sphincter, and the colonic blood supply was examined (Fig. 2B).

At the upper end of the rectal stump, on the right hemicircumference, a row of continuous running sutures was placed between the rectal mucosal layer and all layers of the colon. In the next step, on the left side, an anterior row was also sutured by the same parachuting method as the right row at the level of the dentate line. Avoid suturing the mesocolon to prevent blood circulation obstacle of exteriorized colonic segment (Fig. 2C).

An endotracheal tube was inserted through the exteriorized colonic segment, with the end of the tube reaching the level of the reconstructed peritoneal reflection (Fig. 2E). After that, the tracheal catheter was sutured with the exteriorized colonic segment (Fig. 2F).

### Second stage

The second surgical stage was scheduled about 2 weeks after the first stage. The robust adhesions formed between the colon and rectal stump after approximately 14 days. After most of the colon function was restored, this is the time the fecal drainage tube was removed and the exteriorized colon was transected at the level of the anal canal section. Under spinal anesthesia, the patient was placed in the jackknife position. At the level of the anal verge, the pulled-through colonic segment was excised. After that, the internal anal sphincter was repaired by folding and suturing both ends, and the anal was reconstructed.

### Postoperative management

Intravenous nutrition was administered after first-stage operation until the bowel sounds resume and the first anal exhaust.

Bladder catheter was kept for a week to prevent urinary retention and cystoinflation. Besides, keeping bladder catheter can avoid contamination of the colon stump.

The dressings of anus should be changed daily; in the meantime, viability of exteriorized colonic segment was checked every day to prevent necrosis and pay attention to whether it is retracted. As Fig. 4B shows, the color and luster of the exteriorized colonic segment were ruddy 14 days after 1-stage procedure.

After 7 days of second-stage operation, digital rectal examination should be performed regularly, once every 3–4 days, to prevent the annular stenosis caused by excessive contracture of scar at the coloanal anastomosis.

Two weeks after the second-stage operation, patients were given instructions to perceive contraction and relaxation of the anal sphincter, and to contract while keeping stable abdominal pressure simultaneously, several times a day, for about 3 months, to strengthen the control of the sphincter.

## Result

**Fig. 3:**
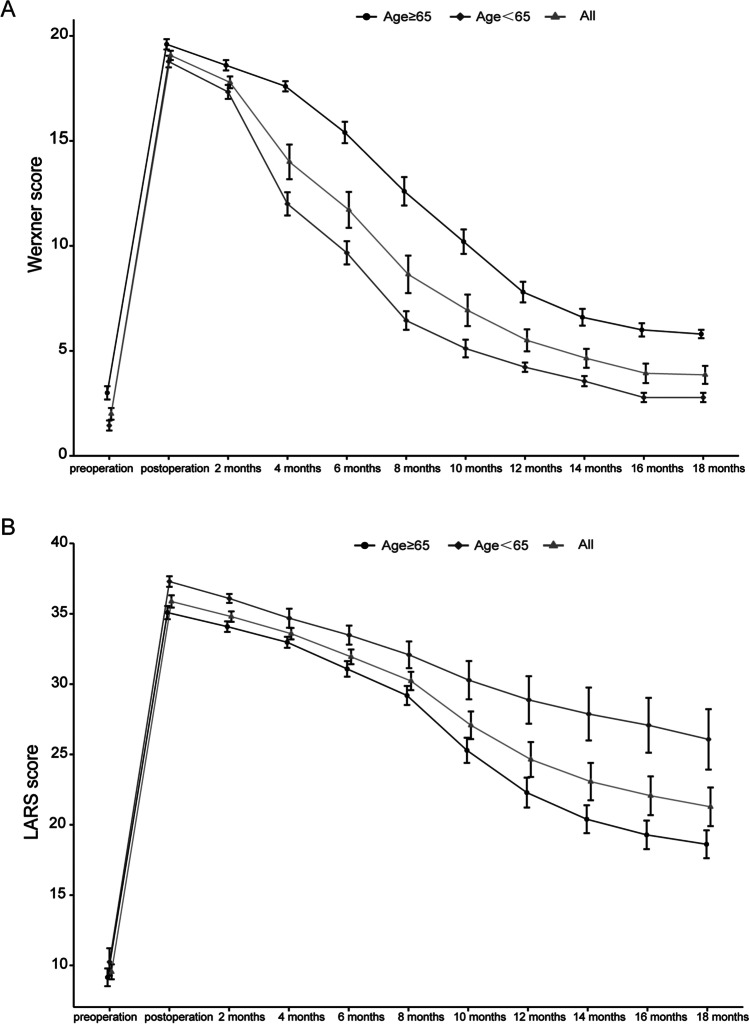
Changes in fecal incontinence scores. The patients were divided into the young and elderly groups. Fecal incontinence score indicates the sum of solid, liquid, gas, wearing pad, and lifestyle alteration scores. The parameters are obtained using the mean/average across all values of patients and the standard deviation of all values around this mean. LARS low anterior resection syndrome

**Fig. 4:**
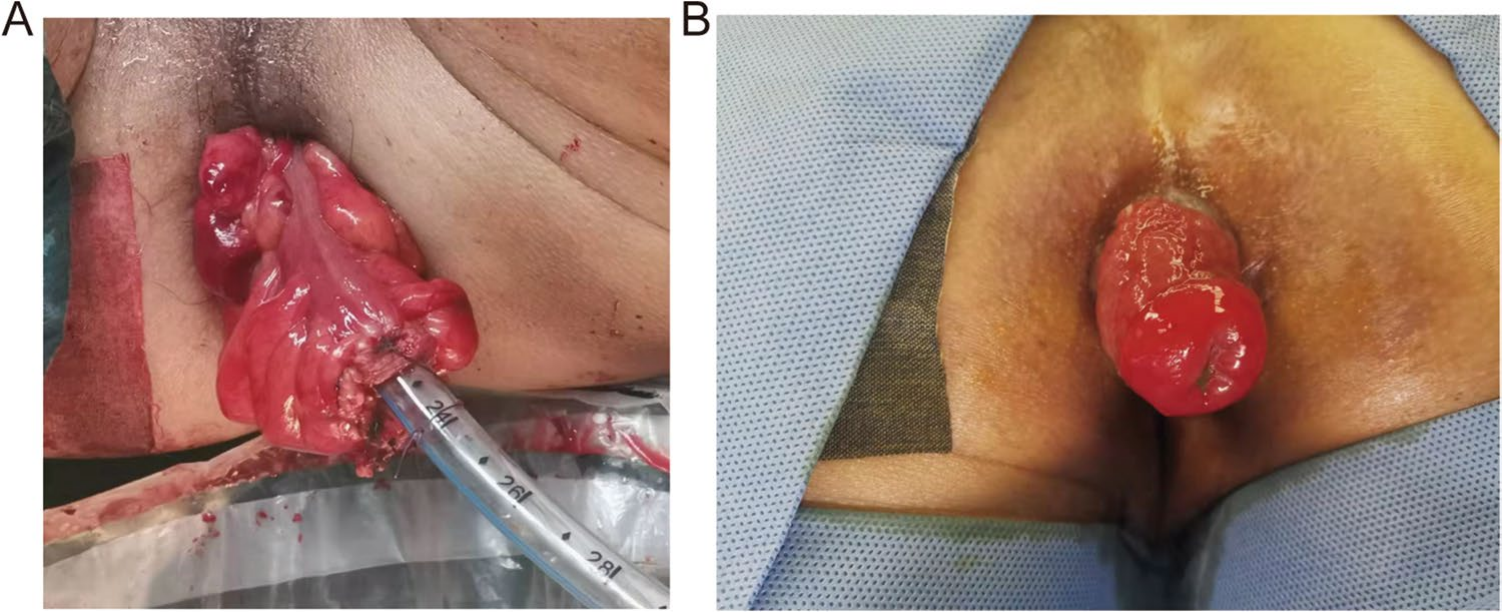
Views of the colonic stump. **A** View of the colonic stump at the end of the 1-stage procedure. **B** View of the colonic stump 14 days after 1-stage procedure

There were 14 patients undergoing operations followed with the above protocol. All procedures were minimally invasive, and all patients signed an informed consent form. Detailed demographic and clinical data are shown in Table 1. The mean (SD) operative time of first stage was 274.5 min (range 220–370) with an average estimated blood loss of 85.9 mL (range 33–200). And the mean (SD) operative time of second was 42.4 min (range 25–76) with an average estimated blood loss of 14.2 mL (range 5–50; Table 2). There were no intraoperative operative complications and only 1 patient (7%) receiving intraoperative transfusions. Four postoperative anastomosis-related complications occurred in 14 patients, including perianastomotic abscess: 1 case (7%), anastomotic stricture: 1 case (7%), and colonic ischemia of the exteriorized colonic segment: 2 cases (14%). The mean (SD) interval between the first stage and second was 14 days (rang 13–16). All patients had an R0 resection without tumor recurrence with an average follow-up time of 14 months. Disease progression and bowel function were followed through serial bimonthly. Bowel function was evaluated using Wexner [17] continence score, known as fecal incontinence score that indicates the sum of solid, liquid, gas, wearing pad, and lifestyle alteration scores. The parameters were obtained using the mean/average across all values of patients and the standard deviation of all values around this mean. The line chart shows the gradual recovery of function of defecation-regulating, which began from 4 months after second operation. After that, the function of defecation-regulating tended to normalization gradually (Fig. 3). We used the LARS score as another measure of bowel dysfunction after surgery [18]. Mean LARS scores and the proportion of patients categorized with none, minor, or major LARS for each group at ≤ 8 months and ≥ 18 months are shown in Table 3 (Fig. 4).

**Table 1:** Pre-operation data

Patients	Sex	Age (years)	Diagnosis	Distance from anal verge (cm)	BMI	Previous anorectal surgery	Neoadjuvant chemoradiotherapy
1	M	74	Rectal cancer	5	18.64	No	No
2	M	59	Rectal cancer	3.5	23.14	No	No
3	F	40	Rectal cancer	5	18.11	No	Yes
4	F	30	Rectal cancer	4.5	17.79	No	No
5	M	61	Rectal cancer	3	26.57	No	Yes
6	F	50	Rectal cancer	3.5	26.71	No	No
7	M	91	Rectal cancer	3	21.09	No	No
8	M	64	Rectal cancer	4	21.72	No	No
9	F	53	Rectal cancer	5	25.53	No	No
10	F	65	Rectal cancer	3	22.34	No	No
11	F	42	Rectal cancer	3.5	24.21	No	Yes
12	M	71	Rectal cancer	2.5	25.31	No	No
13	F	69	Rectal cancer	2	21.65	No	Yes
14	M	55	Rectal cancer	3	20.89	No	Yes

*BMI* body mass index

**Table 2:** Intraoperative data

Patients	Operative time (min)	Blood loss (mL)	Time between 1st and 2nd stage (days)	Intraoperative morbidity	Diverting stoma
1	240 + 58	100 + 20	16	No	No
2	245 + 25	100 + 20	14	No	No
3	300 + 40	33 + 20	14	No	No
4	350 + 35	50 + 10	14	No	No
5	300 + 30	200 + 50	14	No	No
6	370 + 76	100 + 5	14	No	No
7	220 + 64	50 + 5	14	No	No
8	229 + 25	80 + 10	13	No	No
9	285 + 61	100 + 5	13	No	No
10	240 + 30	50 + 10	14	No	No
11	246 + 44	70 + 10	14	No	No
12	273 + 35	100 + 20	13	No	No
13	263 + 28	100 + 10	13	No	No
14	282 + 40	70 + 5	16	No	No

**Table 3:** Distribution of LARS score categories between the two groups at ≤ 8 months and ≥ 18 months

	Overall (*n* = 14)	Age ≥ 65 years (*n* = 5)	Age < 65 years (*n* = 9)	*p*
≤ 8 months				
Mean LARS score, points (SD)	30.4 (2.0)	32.6 (1.3)	29.1 (2.468)	0.029
LARS categories				
No LARS	0 (0%)	0 (0%)	0 (0%)	
Minor LARS	6 (43%)	1 (20%)	5 (56%)	
Major LARS	8 (57%)	4 (80%)	4 (44%)	
≥ 18 months				
Mean LARS score, points (SD)	21.2 (5.1)	26.0 (4.796)	18.5 (2.9)	0.019
LARS categories				
No LARS	8 (57%)	1 (20%)	7 (78%)	
Minor LARS	4 (29%)	2 (40%)	2 (22%)	
Major LARS	2 (14%)	2 (40%)	0 (0%)	

*LARS* low anterior resection syndrome

## Discussion

Despite significant improvement in techniques of surgery and postoperative care, low rectal and coloanal anastomoses still have pelvic-related morbidity such as perianastomotic abscess formation and a non-negligible incidence of anastomotic leakage. This is specifically true for patients who have received neoadjuvant treatment with chemoradiotherapy. Recently, pull-though procedure gained popularity again not only as remedy after anastomotic leakage but also as first treatment of choice in case of very low rectal cancer [16, 19]. In addition, although intersphincteric resection (ISR) had good oncological outcome, anal dysfunction and quality of life (QOL) is a noteworthy problem [20]. The anal function improved gradually following ISR, but the Wexner score at 24 months postoperatively was still very high (7.9 ± 4.1) [21]. In order to prevent the complications of low rectal anastomotic leakage, diverting stoma ileostomy or colostomy is often performed, which still have various complications and impact the quality of life. Besides, partial diverting stoma will evolve into permanent diverting stomas [22].

At present, the commonly used pull-out surgery includes modified Bacon and Turnball-Cutait delayed coloanal anastomosis. The obvious difference between the two operations is that the former requires colonic stump resection and mucocutaneous anastomosis is performed, besides two-stage colonic stump resection, while the latter requires delayed coloanal full thickness suture [12, 13, 23]. The complication associated with this distinction is rectal stump retraction. And we took the parachute intussuscept anastomosis in the first stage. This method of suture not only strengthens the fixation between the colon and rectum, but mimics the Houston’s valve. Simulating the Houston’s valve (rectal wrinkles), the left and right half circles were sutured in parachute style. They mimicked the physiological function of the Houston’s valve and have a stronger fixation ability to delay colon retraction and defecation. Humans have unique anatomical structures called the valves of Houston (called also plica transversalis recti), located in the rectum. A British/Irish anatomist named John Houston first described them in 1830. Permanent, transverse, or semicircular folds are designed to support the weight of feces when they are directed into the rectum, thus slowing the exit [24, 25]. Approximately 15% of the basal anal canal resting tone is generated by the expansile vascular anal cushions, which, along with secondary anal mucosal folds, provide a hermetic seal [26]. As a result, it may prevent or reduce the direct pressure exerted by feces on the pelvic floor during defecation. In addition, the physiological functions of the Houston’s valve also include separation of intestinal gas from feces [27].

Babcock et al. initially proposed the pull-through technique with total internal sphincter cutting off, but Turnball-Cutait abandoned this procedure as it was considered complex and damaged the anal function, because the internal anal sphincter contributes 55% of the maximum resting pressure measured by anorectal manometry, although the external anal sphincter and the hemorrhoid plexus contribute 30% and 15%, respectively [28]. And as a result, the rate of anastomotic stricture and colonic ischemia and necrosis of the exteriorized colonic segment is greatly increased. To improve functional outcome, we performed a modified technique. In our procedure, the dissection of internal anal sphincter can distinctly reduce the contractility and protect the exteriorized colonic segment from ischemic necrosis. Repairing the internal anal sphincter in second stage can effectively preserve anal function. One main reason is that in comparison with subtotal ISR and total ISR, partial excision of the internal sphincter was significantly associated (*P* = 0.04) with better anal function assessed by the Wexner score [29]. Consequently, if only partial sphincter resection or repair after cutting off sphincter, the anal function was assuredly preserved. It has to be said that postoperative functional impairment of intersphincteric resection (ISR) appears to be common, and up to 60% of patients will suffer some degree of fecal incontinence [30]. In patients who accepted our operation, anal function recovered significantly from 4 months post-operation and then even tended to normal level. And there was a high level of patient satisfaction.

The infection of the retrorectal (presacral) space, during the period after first-stage operation, can result in elevated inflammatory markers and fever. The presacral space is drained by the incision of internal anal sphincter. Constant lavage through a catheter usually is effective.

Not all patients’ anal function can recover quickly. Among them, anal function of elderly patients and patients with preoperative anal dysfunction could not reach the normal level after operation. Defects with tissue repair and wound healing ability may be partially responsible. In conclusion, though there was a paucity of high-grade evidence, the described methods can preserve the anal function of patients as much as possible and can become one of the effective methods in the surgical treatment of low rectal carcinoma.

## References

[1] Salerno G (2006). Defining the rectum: surgically, radiologically and anatomically. Colorectal Dis.

[2] Rullier E, Denost Q, Vendrely V, Rullier A, Laurent C (2013). Low rectal cancer: classification and standardization of surgery. Dis Colon Rectum.

[3] Eriksen MT, Wibe A, Norstein J, Haffner J, Wiig JN (2005). Anastomotic leakage following routine mesorectal excision for rectal cancer in a national cohort of patients. Colorectal Dis.

[4] Kitaguchi D (2019). Recurrence of rectal anastomotic leakage following stoma closure: assessment of risk factors. Colorectal Dis.

[5] Paun BC, Cassie S, MacLean AR, Dixon E, Buie WD (2010). Postoperative complications following surgery for rectal cancer. Ann Surg.

[6] Gessler B, Haglind E, Angenete E (2012). Loop ileostomies in colorectal cancer patients--morbidity and risk factors for nonreversal. J Surg Res.

[7] Danielsen AK (2017). Early closure of a temporary ileostomy in patients with rectal cancer: a multicenter randomized controlled trial. Ann Surg.

[8] Babcock WW (1989). William Wayne Babcock 1872-1963. The operative treatment of carcinoma of the rectosigmoid with methods for the elimination of colostomy. Dis Colon Rectum.

[9] Schiessel R (2015). Julius von Hochenegg published the pull-through method for rectoanal reconstruction 125 years ago. Dis Colon Rectum.

[10] Bacon HE (1971). Present status of the pull-through sphincter-preserving procedure. Cancer.

[11] Black BM (1952). Combined abdominoendorectal resection; technical aspects and indications. AMA Arch Surg.

[12] Cutait DE, Figliolini FJ (1961). A new method of colorectal anastomosis in abdominoperineal resection. Dis Colon Rectum.

[13] Biondo S (2020). Two-stage turnbull-cutait pull-through coloanal anastomosis for low rectal cancer: a randomized clinical trial. JAMA Surg.

[14] Jarry J, Faucheron JL, Moreno W, Bellera CA, Evrard S (2011). Delayed colo-anal anastomosis is an alternative to prophylactic diverting stoma after total mesorectal excision for middle and low rectal carcinomas. Eur J Surg Oncol.

[15] Sage PY (2018). Laparoscopic delayed coloanal anastomosis without diverting ileostomy for low rectal cancer surgery: 85 consecutive patients from a single institution. Tech Coloproctol.

[16] Remzi FH, El Gazzaz G, Kiran RP, Kirat HT, Fazio VW (2009). Outcomes following Turnbull–Cutait abdominoperineal pull-through compared with coloanal anastomosis. Br J Surg.

[17] Jorge JM, Wexner SD (1993). Etiology and management of fecal incontinence. Dis Colon Rectum.

[18] Emmertsen KJ, Laurberg S (2012). Low anterior resection syndrome score: development and validation of a symptom-based scoring system for bowel dysfunction after low anterior resection for rectal cancer. Ann Surg.

[19] Hiranyakas A, Ho YH (2011). Laparoscopic ultralow anterior resection versus laparoscopic pull-through with coloanal anastomosis for rectal cancers: a comparative study. Am J Surg.

[20] Shirouzu K, Murakami N, Akagi Y (2017). Intersphincteric resection for very low rectal cancer: a review of the updated literature. Ann Gastroenterol Surg.

[21] Kawada K, Hida K, Hasegawa S, Sakai Y (2018). A comparison of the long-term anorectal function between laparoscopic intersphincteric resection and low anterior resection for low rectal cancer. Surg Today.

[22] Liu J (2021). Nomogram for predicting the probability of permanent stoma after laparoscopic intersphincteric resection. J Gastrointest Surg.

[23] Khubchandani IT (1987). The Bacon pull-through procedure. Dis Colon Rectum.

[24] (1831) Dr. Houston on the mucous membrane of the rectum. Med Chir Rev 14:213–214 PMC5109380 29919884

[25] Shafik A, Doss S, Ali YA, Shafik AA (2001). Transverse folds of rectum: anatomic study and clinical implications. Clin Anat.

[26] Lunniss PJ, Scott SM, Fenner DE, Sultan AH, Thakar R (2007). Perineal and Anal Sphincter Trauma: Diagnosis and Clinical Management.

[27] Heitmann PT (2021). Understanding the physiology of human defaecation and disorders of continence and evacuation. Nat Rev Gastroenterol Hepatol.

[28] Penninckx F, Lestar B, Kerremans R (1992). The internal anal sphincter: mechanisms of control and its role in maintaining anal continence. Baillieres Clin Gastroenterol.

[29] Ito M (2009). Analysis of clinical factors associated with anal function after intersphincteric resection for very low rectal cancer. Dis Colon Rectum.

[30] Tilney HS, Tekkis PP (2008). Extending the horizons of restorative rectal surgery: intersphincteric resection for low rectal cancer. Colorectal Dis.

